# Consensus on core competencies for simulation training in ultrasound-guided renal biopsy

**DOI:** 10.1371/journal.pone.0319788

**Published:** 2025-05-09

**Authors:** Andrea J. Doyle, Colin P. Cantwell, Claire Mulhall, Richard Arnett, Claire M. Condron

**Affiliations:** 1 RCSI SIM Centre for Simulation Education and Research, RCSI University of Medicine and Health Sciences, Dublin, Ireland; 2 St Vincent’s Healthcare Group and UCD college of Health and Agricultural sciences, Dublin, Ireland; 3 Quality Enhancement Office & Health Professions Education Centre, RCSI University of Medicine and Health Sciences, Dublin, Ireland; Affiliated Hospital of Nanjing University of Chinese Medicine: Jiangsu Province Academy of Traditional Chinese Medicine, CHINA

## Abstract

**Rationale and objectives:**

To define the competencies in ultrasound knowledge and skills that are essential for interventional radiology trainees to master when performing ultrasound-guided renal biopsy, and to design simulation-based training to develop these competencies.

**Materials and methods:**

A whole-task training and competency-based assessment methodology was adopted. 42 fellowship-trained interventional radiologists were contacted to participate in the process. An anonymous three-round modified Delphi process was used to identify and assess the crucial procedural steps in performing an ultrasound-guided renal procedure. The primary survey round allowed free text submission of the procedure steps. The primary survey was analysed to identify the common key tasks and these were grouped in procedural domains. For rounds two and three consensus on key steps was evaluated. 75% agreement was required to progress to the final task list. The final task list was reviewed to identify simulation strategies.

**Results:**

Nine interventional radiologists completed the three-round modified Delphi process. 24 competencies were determined to be important across the seven procedural domains. One competency identifying contraindications was removed due to disagreement among participants. The educational review of the final task list identified simulation strategies: three domains involving simulated participants and four domains requiring hybrid simulation involving simulated participants, simulator training and interprofessional simulation.

**Conclusion:**

Twenty-three competencies were identified in a blueprint for the development of a simulation curriculum to meet the training requirement of radiologists performing ultrasound-guided renal biopsy.

## Introduction

Ultrasound-guided renal procedures are widely performed by an interventional radiologist and are an effective and minimally invasive alternative to conventional surgery. Ultrasound-guidance is used extensively in procedures including focal and non-focal renal biopsy, cyst aspiration, nephrostomy catheter insertion, and renal tumour ablation. The advantages and benefits of this ultrasound-guided approach include a reduction in complications (including infection and blood loss), less pain (due to the small incision sites), patient convenience (minimally invasive, and often an outpatient procedure) and lower costs [1]. However, these benefits are only assured if the interventional radiology (IR) services have a highly trained workforce.

The success of IR procedures relies on the interventional radiologist’s ability to interpret ultrasound images in real-time to guide and manipulate a needle in the body to a target [2]. All ultrasound-guided procedures in the kidney require significant dexterity and cognitive decision-making skills to manage the ultrasound image, identify the target, identify the non-target organs and obstructions, optimise image quality, mitigate image artefacts, and guide the applicator or needle to the correct location in the body [3].

There is an identified need to develop simulation-based training to assist with training interventional radiologists to perform renal procedures [4]. There is an increasing demand for trained interventional radiologists in the UK and Ireland to keep staffing levels in line with European averages [1,5]. In 2020, a European-wide needs assessment was conducted to reach consensus on the technical procedures in radiology to include in simulation-based training for residents. A Delphi working group of international radiology and medical education experts highlighted the need for basic ultrasound operation as the top ranked procedure for simulation-based training, with ultrasound kidney imaging and ultrasound guidance for interventional procedures ranking third and sixth respectively [4].

The optimal implementation of simulation-based medical education (SBME) remains poorly reported and while best practice is yet to be defined, studies have begun to characterise and evaluate factors that would impact the quality of SBME curricular implementation [6,7]. The need for training identified through problem identification and a general needs assessment is recognised as the first step in the systematic design in curricular development [8]. A needs assessment involving stakeholders with contextual expertise is pertinent to problem identification and definition, and in distinguishing the critical clinical competencies associated of the intended intervention [9]. In this study, we employ a modified Delphi consensus process to define the critical procedural steps associated with ultrasound guided kidney interventional radiology biopsy procedures. Though the consensus process we aim to define the competencies in ultrasound knowledge and skills that are essential for trainees to master performing ultrasound-guided renal biopsy.

## Materials and methods

This study was approved by the RCSI University of Medicine and Health Sciences Research Ethics Committee REC202207005.

### Steering group

A steering group was formed with members with expertise in interventional ultrasound, medical education, simulation and curriculum development. AJD convened the steering group and led the modified Delphi investigation with collaboration from other authors (CPC, CM, RA, and CMC).

### Participant recruitment

Participants were canvased to enrol in the study via email through the national network of fellowship trained IR consultants, the Irish Society of Interventional Radiology (https://isir.i.e.,) their participation was voluntary and their anonymity was maintained throughout the process. Participants were recruited from 22^nd^ November through to 22^nd^ December 2023. Participants were provided with an information leaflet when they were invited to participate. On conformation of their interest to participate, participants were sent an electronic consent form to complete and sign before the study began. CM acted as a gatekeeper for participant data and corresponded with the participants through email and de-identified the study data before other members of the steering group accessed data.

### Study design

**Fig 1:**
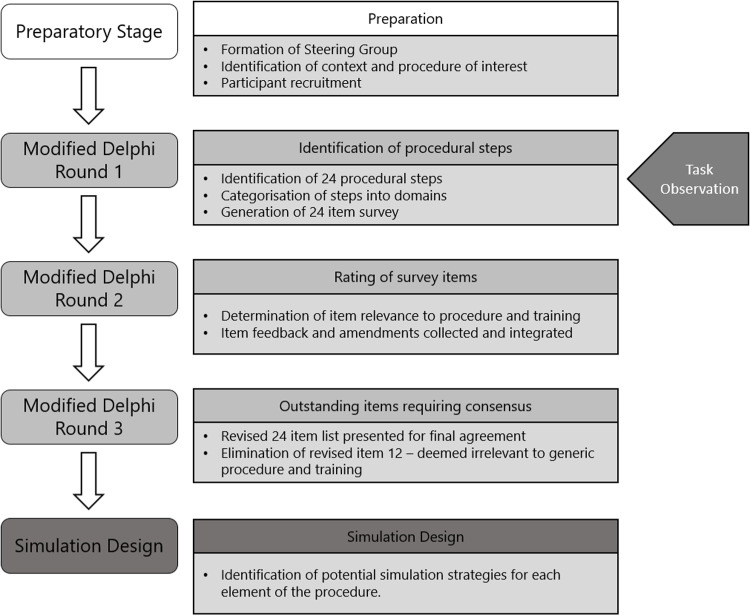
Overview of the modified Delphi process including task observation, data collection and analysis summaries.

Modified Delphi [10] and task analysis [11] processes were integrated in this study to identify the critical procedural steps associated with ultrasound guided kidney biopsies, see Fig 1. This dual approach enabled the lead investigator (AJD) to better understand the contextual information returned in each round of the modified Delphi process, by observing ultrasound guided kidney biopsy procedures at a clinical site.

Participants were not required to convene in person; instead, their anonymous responses were shared with the group in subsequent rounds for further exploration until a consensus was achieved. Data collection was performed using an online Microsoft Form. The initial list of procedural steps was generated by the panellists in the first round of data collection, in subsequent rounds these list items were rated in terms of their importance, modified for utility, and categorised into domains [12]. This data was then presented to the participants in subsequent rounds, and participants expressed their level of agreement concerning the significance of each item in relation to the procedure and consequently training, choosing from ‘Strongly Disagree,’ ‘Disagree,’ ‘Neither Agree nor Disagree,’ ‘Agree,’ and ‘Strongly Agree.’ An a priori threshold of 75% agreement between panellists was established [13]. The identified procedure domains were further interrogated by the research team to determine potential simulation strategies that could be incorporated into a SBME training intervention.

### Data collection and analysis

#### Round 1.

The first Delphi round presented panellists with an open-ended question, designed to capture their collective expertise and identify the skills required to perform ultrasound guided kidney biopsy.

*“Your patient has arrived in the radiology suite for a scheduled renal focal lesion biopsy interventional radiology procedure. Please describe the process for an ultrasound guided interventional radiology procedure of the kidney.* ***In as much detail as possible****, please include the steps you would take and the order in which you do them. There is no limit to the length of your response.”*

Panellists were encouraged to provide free-text descriptors and there was no word limit associated with their responses. During this first Delphi round, ultrasound guided kidney procedures performed by IR consultants at clinical sites were observed by AJD. Data from the first Delphi round captured the panellists’ approaches to the kidney biopsy procedure, and were grouped and sequenced (see Appendix) by AJD with support of CPC, a Consultant Interventional Radiologist and CMC, Director of Simulation Education.

#### Round 2.

In the second Delphi round, panellists were then asked to review the list of items generated in the previous round, and were required to report their agreement regarding the importance of each listed item in terms of its relevance to the procedure. Participants indicated their agreement level regarding the importance of each item concerning the procedure, and thus, training, by selecting from ‘Strongly Disagree,’ ‘Disagree,’ ‘Neither Agree nor Disagree,’ ‘Agree,’ and ‘Strongly Agree.’ Additionally, participants contributed supplementary information they considered essential by utilizing free-text boxes provided at the conclusion of each section in the Microsoft Form.

#### Round 3.

The items that reached consensus as important in round 2 were included in the third Delphi round, however participants were only required to rate an outstanding item. After this final Delphi round the research team, including RA, Director of Psychometrics, reviewed the data and finalised the competency list.

#### Simulation design.

The final competency list was reviewed by the steering group. The group specified the simulation based educational training intervention that is the most effective simulation strategies for each element of the procedure.

## Results

Of the 42 interventional radiologists invited to complete the process, nine completed the three-round modified Delphi process (21%). The median participant age was 51 (34–63) years, with years of professional practice (outside of training) ranging from ≤1–28 years. Table 1 presents the demographic profiles of the participants in the Delphi process.

**Table 1:** Participant demographic information.

Demographics	Total *(n=9)*
Gender	
Male	9
Female	0
Age range	
18-35	2
36-50	4
51-70	3
Years of professional practice *(outside of training)*	
≤1 year	3
2-10 years	1
11-20 years	3
21-30 years	2
Involvement in Radiology Education	
Yes	6
No	3

### Round 1.

During the initial Delphi round, AJD observed three ultrasound-guided kidney procedures conducted by IR consultants. AJD documented each step as the consultant verbally outlined their approach and organized the procedural tasks in sequence. Clarifying questions were posed to gain insight into the event sequencing and to observe the interactions between IR consultants and other healthcare professionals participating in the procedure. Using descriptive analysis, the task observation data was integrated with the first-round data, and supported the categorisation of a list of items into domains for ultrasound guided kidney biopsy procedures.

The 24-item list categorised into seven domains generated in round 1 is included in the Appendix. This categorised list was sent to the participants in the second round.

### Round 2.

In the second Delphi round, panellists reviewed the 24-item list. The results of participant rating that in round two are presented in Table 2. 23 items reached consensus, with 75% agreement, with minor additional amendments suggested from participants.

**Table 2:** Participant agreement Delphi Round 2 and Round 3.

Domain	Survey Item	Round 2 % agreement	Round 3 % agreement
Review History, Chart & Imaging	Review patient chart Name DOB, patient info, bloods, fasting, allergies. Review request/indications (N.B. which kidney) Review patient’s history, e.g., any history of prior focal lesions, renal tract infection or malignancy. Review patient medications especially antiplatelet, anticoagulants or other agents increasing bleeding risk and ensure agents stopped at an appropriate interval pre-procedure.	89	–
	Ensure the patients has received their normal medications otherwise, e.g., antihypertensive.	78	–
	Review all available relevant imaging and plan likely approach. Consider: Is there bowel? Is there a safe route? How vascular is lesion?	100	–
	Choose/propose guidance technique, i.e., CT or ultrasound?	89	–
Consult & Consent	Consent the patient (ideally preformed much earlier than immediately prior to the procedure) Meet and identify the patient. Explain the procedure. Inform patient of alternatives as well as risks and benefits of procedure. Offer patient opportunity to ask any questions they might have.	100	–
	Assess Patient’s general fitness: can patient lie prone or will a decubitus approach be required?	100	–
Prepare Interventional Radiology Suite & Communicate with Colleagues	Discussion with nursing and radiography staff regarding proposed procedure, patient positioning and any special/non-standard considerations.	100	–
	Time out standard questionnaire and review with patient in room, including baseline observations, antibiotics, and sedation/pain relief.	100	–
	Confirm IV access for sedation.	100	–
	Gather all necessary equipment with team including needle, coaxial needle, appropriate biopsy tray, aseptic technique materials, and local anaesthesia and biopsy instruments.	100	–
	Ask nursing staff to commence monitoring; pulse, blood pressure and O2 saturations at a minimum. Optimally, cardiac trace in addition.	100	–
	If the patient’s blood pressure exceeds 160/100mmHG after conscious sedation and antihypertensive medications, postpone the procedure and seek a medical review.	**67**	**67**
Patient Positioning	Ask the patient to adopt the optimal position to approach the lesion in question **OR** place the patient into prone position with assistance from team.	100	–
	Perform a planning ultrasound: Select curvilinear probe. Confirm lesion is visible on ultrasound. Confirm lesion size and location. Ensure the planned approach is feasible ◊ Decide if biopsy is possible under ultrasound; will CT be required? Identify any at risk non-target structures. Determine skin site for puncture. Awareness that the kidney will rise superiorly underneath the diaphragm once conscious sedation is administered; this may affect the point of insertion of the needle.	100	–
Procedural Preparation & Ultrasound Optimisation	Sterile prep, skin drape and ultrasound probe cover.	100	–
	Optimise Ultrasound machine parameters: Choose curvilinear probe Find orientation of probe and image by tapping on transducer array Scan region of interest to locate kidney and lesion of interest Move focus to region of interest in the image Adjust depth of image to lesion Adjust TGCs for uniform image speckle Modify gain to enhance biopsy and surrounding soft tissue contrast Modify frequency to optimise image quality Measure distance from skin to lesion to inform approach and trocar selection Modify approach and patient position as necessary	100	–
Biopsy Procedure	Administer Sedation + Analgesia: Local anaesthetic with 1% lidocaine Administer conscious sedation (midazolam +/- fentanyl IV) and titrate thereafter according to need	100	–
	Needle position Skin nick with 11 blade on surface of the skin for entry point of needle Advance needle into the lesion in the kidney under direct US guidance, anaesthetising the tract from skin to renal capsule Confirm needle position on ultrasound image. Ensure there is a safe 2 cm ‘throw’ for the biopsy needle and then obtain at least 2 good quality cores using selected needle. Remove central stylet and ensure there is no bleeding Introduce matched gauged biopsy needle via the coaxial needle	78	–
	Biopsy cores Take 1,2 or 3 cores (depending on incidental bleeding) varying the angle between cores, under direct ultrasound visualisation Cores placed in fluid (saline/formalin) histology specimen container and patient’s details double-checked on the container. Ensure samples satisfactory. Examine for presence of white or grey material as opposed to pink or red material. Label and send sample to lab; if urgent consider hand delivery of sample.	100	–
	After Biopsy If morphological position of lesion and approach are high risk for bleeding than plug tract with gel foam pledget Remove coaxial needle. A focussed post procedural ultrasound will then be performed to look for any possible post procedural complications such as a large hematoma. Pressure applied to the puncture site, dressing applied.	89	–
Post Procedure	Details of procedure and patient’s condition handed over to staff receiving back the patient and patient then transferred back to ward The patient is then observed post procedure: Analgesia PRN.	100	–
	Report of procedure written up and standing orders prescribed	100	–
	Patient counselled re post biopsy precautions	100	–
	Answer patient questions	100	–

Item 12 from the domain Prepare Interventional Radiology Suite & Communicate with Colleagues caused dissensus, related to local policies. One participant shared the following rationale for disagreeing with this statement, *“If blood pressure is too high, I would not waste time trying to get it to a normal level. I would discuss with referring doctor and just postpone the procedure until they can optimise the BP” [P4].* Another commented *“High BP has always dropped with sedation” [P4].* CPC the expert IR consultant in the steering group, advised this statement should be revised based on participant feedback and was changed to “*If the patient’s blood pressure exceeds 160/100mmHG after conscious sedation and antihypertensive medications, postpone the procedure and seek a medical review.”* CPC also advised this is not integral to the IR kidney biopsy procedure and if dissensus remained after the third round of data collection, this item could be removed. The updated item list presented in table 2, revised to include participant feedback, was sent to the participants in the third round.

### Round 3.

Participants were presented with the revised competency list for final agreement and to capture outstanding feedback. Agreement was reached on 23 items. one outstanding item, item 12 (see Table 2), did not receive consensus and was removed. There was no additional free text feedback.

Results from each round of the modified Delph process and the amendments made at each stage are included in Appendix.

### Simulation design.

AJD & CMC with the support of CPC, identified the procedure domains that could be incorporated into a simulation based educational training intervention, identifying the most effective simulation strategies, see Table 3.

**Table 3:** Simulation Strategies for each procedural domain.

	Simulated Participants	Simulator Trainer	Interprofessional Simulation	*Hybrid Simulation
**Review History, Chart & Imaging**	✓			
**Consult & Consent**	✓			
**Prepare Interventional Radiology Suite & Communicate with Colleagues**	✓	✓	✓	✓
**Patient Positioning**	✓	✓	✓	✓
**Procedural Preparation &** **Ultrasound Optimisation**	✓	✓	✓	✓
**Biopsy Procedure**	✓	✓	✓	✓
**Post Procedure**	✓			

*Hybrid Simulation refers to the combination of simulator trainer and simulated participant approaches.

## Discussion

There is a need to train large numbers of radiologists to deliver radiology procedures. The UK and Ireland are experiencing a rising demand for well-trained radiologists to align staffing levels with European averages [1,5]. A 2017 report from the Royal College of Radiologists (RCR) indicated a widening gap between the supply and demand for radiology services. The shortage of 1,000 consultants in 2017 was projected to grow to approximately 1,600 consultants by 2022, posing a significant challenge to radiology services.

Radiology training currently follows the apprenticeship model, which requires significant resources, including mentored training time, may result in longer operative times, and has the potential harm to patients. Simulation-based medical education (SBME) successfully contributes to quality health professions education [14] and radiology training [15]. It is an effective approach to acquire, retain and transfer skills to clinical practice, at a minimum equivalent to that achieved through the traditional apprenticeship model [16]. SBME in radiology training is cost effective [17], improves trainee’ skills [18] and significantly improves patient outcomes through reduction in infections [19], fewer errors and less time on the procedure [20].

Many studies have reported positively on the role of SBME in sonography, noting the benefits of this type of training; the reduction of stress and risk to the patient and improvement in skill acquisition and procedural technique in an unpressurised or “lower stakes” environment [21,22]. In this lower stakes environment, SBME contributes to the psychological aspects of IR procedure performance, including increased confidence and reduced anxiety in trainees [23].

The goal is to prepare individuals not only for the specific context training but also to enable the transfer of acquired skills to real-world scenarios [24]. While the RCR IR curriculum includes simulation as a teaching and learning methodology [25], there is no guidance on how best to implement SMBE and what approaches should be considered.

The design and implementation of SBME should address the current training needs. Often individual clinical skills are developed in isolation on task trainers in low fidelity environments. Whole-task training recognizes that effective learning involves more than just mastering individual components in isolation. It emphasizes the interconnectedness of various skills and the need to develop a holistic understanding of tasks or challenges. We must consider the full spectrum of skills that are required, as opposed to those specifically related to model based procedural activities. It is specifically important in this context for radiology trainees who are intrinsically motivated to become clinically competent [26], and whole-task training will prepare trainees for their clinical role to meet the current staffing demand, and to provide the opportunity to practice and hone the full spectrum of skills associated with a procedure.

There are a range of strategies that can be incorporated into SBME interventions including part task simulation trainers to develop and hone specific technical components of clinical tasks, high-fidelity scenarios including multiple professionals, working with simulated participants to develop communication skills, and virtual and augmented reality training. Research suggests the benefit of whole-task training, especially in routine procedures such as kidney biopsy, when the discrete clinical steps are highly integrated [27]. Whole-task training is a comprehensive approach to learning that encompasses the integrated acquisition of knowledge, skills, and attitudes. In health professions education this method goes beyond isolated procedural skill development and focuses on the coordination of qualitatively different constituent skills.

Whole-task training and competency-based assessment in health professions education relies on simulation as an approximation of clinical practice in which to develop, hone and master procedural skills. A notable finding was the similarities in the descriptions of the procedure from all nine participants, which facilitated the initial identification of the 24 items. This suggests that whole-task training would be a suitable strategy for this type of training as the participants delineated discrete clinical steps that were highly integrated [27].

The main challenge associated with the consensus building activity in this study was related to individual organisational procedures and local practice. After three rounds of data collection, item 12 *If the patient’s blood pressure exceeds 160/100mmHG after conscious sedation and antihypertensive medications, postpone the procedure and seek a medical review* was removed from the itemised list as it had not reached the consensus threshold. Additionally, item 21 was revised to remove specific observation frequency and vital sign limits. While local context and adherence to local guidelines is an important aspect of training for IR trainees at a specific clinical site, ultimately these factors were deemed irrelevant to a national procedural description by the steering group.

Hybrid simulations, involving simulated participants and technology would facilitate whole-task training and an evaluation of competence. Hybrid simulations also emphasize the importance of all of the skills in a particular procedure; 1) those associated with the instruments and imaging technologies, and 2) those associated with patient consultation, interprofessional collaboration, communication and teamwork. The latter grouping of skills historically referred to as “bedside manner” is routinely taught in isolation during undergraduate training and is not routinely incorporated in training in clinical practice [28].

A limitation of the study was the potential gender bias associated with an all-male Delphi panel. The authors extended the initial recruitment period to try to include additional perspectives, however the documented gender imbalance in academic medicine, especially in procedure-oriented fields such as interventional radiology resulted in all male participants [29,30]. This study was also limited in that it was not designed to be an international survey, it was an all-Ireland survey to address a national need for formalised competency-based training. Finally, there was a low response rate to the survey, 21% of the network of Interventional Radiologists engaged with the consensus building activities. While this appears to be a low response rate, it is in line with surveys of this kind, and speaks to the demands and workload of the profession; the European Society of Radiology (ESR) and the Cardiovascular and Interventional Radiological Society of Europe (CIRSE) developed an online survey in 2019, and the response rate was 8.3% [31].

## Conclusion

In conclusion, this Delphi process has led to the identification of 23 competencies in a blueprint deemed essential for the development of a curriculum tailored to the training needs of radiologists performing ultrasound-guided renal biopsy. This blueprint offers a structured approach to integrating simulation-based education into radiology training programs.
